# Organisation d´une campagne de masse et gratuite de distribution des moustiquaires imprégnées d´insecticides à longue durée d´action dans le contexte de la COVID-19 au Niger

**DOI:** 10.11604/pamj.2021.38.119.26664

**Published:** 2021-02-03

**Authors:** Fatima Aboubakar, Hadiza Jackou, Blanche-Philomene Melanga Anya, Boubé Hamani, Patrick Katoto, Charles Shey Wiysonge

**Affiliations:** 1 Bureau Pays, Organisation Mondiale de la Santé, Quartier Plateau, Avenue Mohamed VI 1204, Niamey, Niger,; 2 Programme National de Lutte Contre le Paludisme, Ministère de la Santé Publique, Place des Ministères, BP 623, Niamey, Niger,; 3 Centre for Infectious Diseases, Faculty of Medicine and Health Sciences, Stellenbosch University, Francie van Zijl Drive, Tygerberg 7505, Cape Town, South Africa,; 4 Centre for Tropical Medicine and Global Health, Faculty of Medicine, Catholic University of Bukavu, Bugabo 02, Bukavu, Democratic Republic of Congo,; 5 Department of Global Health, Faculty of Medicine and Health Sciences, Stellenbosch University, Francie van Zijl Drive, Tygerberg 7505, Cape Town, South Africa,; 6 Cochrane South Africa, South African Medical Research Council, Francie van Zijl Drive, Parow Valley 7501, Cape Town, South Africa,; 7 School of Public Health and Family Medicine, University of Cape Town, Anzio Road, Observatory 7935, Cape Town, South Africa

**Keywords:** Malaria, prévention, MILDA, pandémie, SARS-CoV-2, Malaria, prevention, LLIMNs, pandemic, SARS-CoV-2

## Abstract

Les moustiquaires imprégnées d´insecticides à longue durée d´action (MILDA) sont nécessaires dans la lutte anti vectorielle du paludisme. Cependant, leur distribution n´est pas encore optimale en région sub-saharienne. Selon les projections, la pandémie de COVID-19 retarderait davantage la distribution des MILDA. Au Niger, une campagne de distribution des MILDA avec une approche multi-sectorielle (Etat-partenaire-société civile) a été organisée dans le respect de mesure barrières contre la COVID-19. La stratégie porte-à-porte dans les ménages a été retenue pour l´organisation de cette campagne pour éviter les espaces clos et garder l´aspect communautaire. Un total de 13,994,681 personnes a bénéficié de MILDA (soit un taux de réussite de 101%) dans six régions ciblées. Un effort collectif est nécessaire pour pérenniser la lutte contre le paludisme dans l´ère COVID-19.

## Abstract (en)

Long-lasting insecticidal mosquito nets (LLIMNs) are needed for malaria vector control. However, their distribution is not yet optimal in sub-Saharan regions. According to projections, COVID-19 pandemic will further delay the distribution of LLIMNs. In Niger, a distribution campaign of LLIMNs with a multi-sectoral approach (state-partner-civil society) was organized in compliance with barrier measures for preventing transmission of COVID-19. A door-to-door strategy was chosen to implement this campaign, in order to avoid entry into confined spaces and to engage community. A total of 13,994,681 people received LLIMNs (reflecting a success rate of 101%) in six targeted regions. A collective effort is needed to sustain the fight against malaria in the COVID-19 era.

## Commentaire

En dépit d´énormes progrès enregistrés ces dernières années en matière de lutte contre le paludisme, il reste un réel problème de santé publique au Niger. La situation demeure préoccupante dans la mesure où le rapport mondial sur le paludisme de 2019 de l´organisation mondiale de la santé (OMS) classe le Niger parmi les six pays africains qui enregistrent à eux seuls plus de 50% des cas et décès dus au paludisme au niveau mondial [1]. La couverture universelle des populations en MILDA est une stratégie de prévention recommandée par l´OMS dans les programmes de contrôle du paludisme. Cependant, selon ce rapport 2019 de l´OMS, le taux de couverture n´a que très peu augmenté depuis 2015 et il s´est même stabilisé depuis 2016 avec une augmentation très modeste au cours des trois dernières années restant bien loin de l´objectif de couverture universelle.

Depuis 2014, le Niger organise des campagnes de masse de distribution des MILDA en complément du programme de routine avec l´appui de ses partenaires dont l´OMS. Initialement prévue du 17 au 20 avril 2020, cette campagne a été reportée du fait du contexte de la COVID-19. En effet, depuis le 19 mars 2020 date d´enregistrement du 1^er^ cas de COVID-19 au Niger, les campagnes de masse programmées pour la période considérée ont été reportées pendant une durée de plus de trois mois du fait de l´observance des mesures restrictives décrétées par le Gouvernement. Cette suspension temporaire d´activités concerne la tenue des réunions, des ateliers, des formations et des déplacements entre la capitale et les 7 autres régions du Niger.

### COVID-19: un vrai frein aux mesures de lutte contre le paludisme

Les récents succès dans la lutte et l´élimination du paludisme ont réduit le fardeau mondial du paludisme, mais ces progrès sont fragiles et ont vu leur ralentissement durant les dernières cinq années [2]. La rétrogradation des interventions réussies entraîne souvent une résurgence rapide du paludisme, menaçant principalement les jeunes enfants et les femmes enceintes. La situation de la pandémie de la COVID-19 a affecté la mise en œuvre des activités préventives et curatives se traduisant du coup par une augmentation du nombre de cas de paludisme. Ces perturbations associées à la situation de pluviométrie exceptionnelle ayant occasionnées des inondations cette année 2020, pourraient doubler le nombre de décès dus au paludisme chez les jeunes enfants Nigériens dans l´année à venir et pourraient avoir un impact sur les efforts de contrôle de la propagation de la résistance aux médicaments. Une récente modélisation [3] a fait remarquer que dans des régions à forte prévalence de VIH, Tuberculose et Malaria et sur les cinq prochaines années, l´avènement de la COVID-19 entrainerait respectivement une augmentation de 10%, 20% et 36% de ces maladies. Pendant que la modélisation estime que le plus grand impact pour le VIH proviendrait de l´interruption du traitement antirétroviral, pour la tuberculose (TB), il proviendrait de la réduction du diagnostic et du traitement opportuns des nouveaux cas. Concernant le paludisme, l´impact pourrait résulter de l´interruption ou du retard dans l´organisation des campagnes de distribution gratuite des moustiquaires prévues. De surcroit, ces perturbations pourraient entraîner une perte d´années de vie sur cinq ans de même ampleur que l´impact direct de la COVID-19 dans les régions à forte prévalence du trio VIH-TB-malaria. Par ailleurs, la suspension ou le report des campagnes de distribution des MILDA et un recul de 75% de l´accès aux antipaludéens efficaces pourraient selon l´OMS [3] occasionner en 2020 près de 769.000 décès liés au paludisme en Afrique subsaharienne soit le double des chiffres enregistrés en 2018 dans la région, ce qui approchera des taux de mortalité due au paludisme jamais enregistrés depuis 20 ans.

### Mise en place d´un plan adapté à l´ère de la COVID-19: motivation et stratégies

La pandémie de COVID-19 met à rude épreuve la résilience de systèmes de santé solides partout dans le monde. Tenant compte de la fragilité des infrastructures sanitaires en Afrique sub-saharienne, l´OMS souligne qu´il est capital de continuer à prévenir, à détecter et à traiter le paludisme. Pour alléger la charge des systèmes de santé, il est important de garantir l´accès aux mesures essentielles de prévention du paludisme, telles que les mesures de lutte antivectorielle incluant la distribution des MILDA. Au Niger, grâce à l´amélioration de la situation de la COVID-19 conduisant à l´allègement des mesures restrictives et à l´adoption des directives de l´OMS pour la continuité des services de lutte contre le paludisme dans le contexte de la COVID-19, le programme national de lutte contre le paludisme (PNLP) a pris le challenge de conduire une campagne gratuite de distribution des MILDA du 05 au 10 juin 2020 en respectant les mesures de protection individuelles et collectives contre la maladie à COVID-19.

Cette campagne de masse et gratuite de distribution de huit millions des MILDA s´est déroulée dans six régions à fort risque de paludisme à savoir Dosso, Diffa, Maradi, Tahoua, Tillabéri et Zinder. Pour tenir compte des mesures barrières contre l´infection à COVID-19 et du contexte sécuritaire alarmant dans les régions de Diffa et Tillabéri, le PNLP a revu le programme de la campagne ainsi que le calendrier de mise en œuvre. Ainsi, les micro-plans de l´ensemble des 759 centres de santé intégrés (CSI) relevant des quarante-quatre (44) districts sanitaires (DS) concernés ont été révisés pour prendre en compte les outils de prévention de la COVID-19. Le protocole et les outils de la campagne ont également été mis à jour avec conception d´un aide-mémoire adapté pour la formation des agents impliqués. Le plan de communication a été adapté à travers le développement des messages (messages et spots publicitaires radio télévisés, Kakemonos, affiches etc.) et supports éducatifs, dans les trois principales langues parlées au Niger.

**Figure 1:**
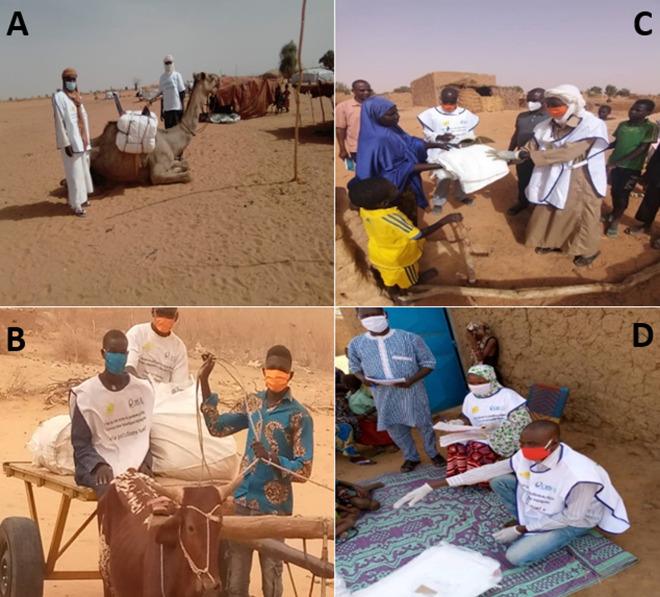
campagne de distribution des moustiquaires imprégnées d’insecticides à longue durée d’action adaptée au contexte avec respect des mesures de protection contre la COVID-19 (Niger, du 05 au 10 juin 2020)

Le PNLP a mobilisé auprès du Fonds Mondial 8.005.656 de MILDA pour une population cible estimée à 13.994.681 de personnes à risque. Les ressources humaines mobilisées et formées pour le besoin de l´activité se composent de 31 membres du comité national d´organisation de la campagne, des équipes cadres des directions régionales de la santé, des DS et CSI ainsi que des chefs religieux, coutumiers et élus locaux à charge de l´organisation, et de la supervision de la campagne, 7.410 relais mobilisateurs et 6.814 agents recenseurs/distributeurs. Les moyens de protection contre la maladie à COVID-19 (bavettes, gants, savons et gels hydro alcoolique) ont été utilisés et le lavage systématique des mains a été respecté au cours de cette campagne. La mise en place des intrants s´est déroulée du 05 au 27 mai 2020 à travers plusieurs canaux dont les camions, les charrettes, les chameaux, des pirogues selon les localités (Figure 1). Pour une bonne mobilisation sociale un lancement national a été effectué le 05 juin 2020 à Niamey sous le haut patronage du Ministre de la santé publique du Niger, Dr IDI ILLIASSOU Mainassara en présence de la Représentante de l´OMS, Dr ANYA Blanche suivi d´un lancement dans chacune des six (6) régions ciblées. La distribution proprement dite des MILDA s´est déroulée du 05 au 10 juin 2020 à travers la stratégie porte à porte en lieu et place de la fixe habituelle.

### Le secret est dans l´Ubuntu

L´Ubuntu est un concept/philosophie sud-africain qui reflète la lutte du Prix Nobel Nelson Mandela et signifie: « la croyance en un lieu universel de partage qui relie toute humanité » ou mieux « je suis parce que tu es ». Comme l´indique le Tableau 1 ci-dessous indiqué, 13.994.681 personnes à risque ont bénéficié des MILDA lors de cette campagne de distribution gratuite soit une couverture globale de 101%. Ce résultat satisfaisant a été atteint grâce à l´adoption de meilleures pratiques portant sur la bonne coordination à tous les niveaux, l´implication des autorités administratives, religieuses et coutumières, la disponibilité des ressources; le respect des mesures barrières contre la COVID-19 et de sécurité par les acteurs, la contribution locale de certaines mairies, l´engouement et l´adhésion totale de la population, le plaidoyer et la mobilisation sociale réussie. Au-delà de cette mobilisation collective, des activités de coordination, communication, formation des acteurs, supervision et évaluation des résultats ont été menées en cascade à tous les niveaux et ont permis de suivre le déroulement de l´activité et de corriger les insuffisances ayant permis d´améliorer la qualité et la performance de la campagne.

**Tableau 1:** couverture en moustiquaires imprégnées d´insecticides à longue durée d´action dans six régions à forte prévalence du paludisme au Niger après une campagne de distribution adaptée dans le contexte de la COVID-19 du 05 au 10 juin 2020

Régions	MILDA Distribuées	Population Bénéficiaire	Couverture Universelle	Ménages Couverts
Diffa	435.813	762.742	103%	129.278
Dosso	1.520.276	2.733.864	100%	463.367
Maradi	1.525.000	2.689.766	102%	455.893
Tahoua	1.043.644	1.916.851	98%	324.890
Tillaberi	2.085.677	3.626.218	104%	614.613
Zinder	1.252.583	2.265.240	100%	383.939
TOTAL	7.862.993	13.994.681	101%	2.371.980

MILDA : moustiquaires imprégnées d´insecticides à longue durée d´action

### Conclusion

L´association COVID-19-malaria peut être dévastatrice, en particulier dans les pays à revenu faible. En perspective, la préparation est donc capitale pour prévenir les effets indirects à court et à long terme de la pandémie à COVID-19, sur les programmes de lutte contre le paludisme et sur les systèmes de santé des pays où les deux maladies peuvent cohabiter. Au Niger, 1) les régions à forte prévalence du paludisme ont constitué la priorité de la campagne; 2) tous les secteurs de la société, y compris les gouvernements, les donateurs, le secteur privé et les organisations de la société civile, ont travaillé ensemble pour garantir la réussite de la distribution des MILDA. 3) Un moyen financier bien défini et tenant compte du défi que pose la COVID-19 fut alloué et de manière efficace pour exécuter la campagne; 4) une cascade de réunions de suivi et évaluation avec souvent utilisation de e-plateforme ont été effectuées. 5) De plus, le PNLP a poursuivi avec une évaluation nationale de la campagne (30 août 2020) en présence des acteurs nationaux, régionaux, sous régionaux et des partenaires et; 6) a continué à tous les niveaux la sensibilisation de la population pour l´utilisation effective des MILDA distribuées. Ce faisant, le Niger envisage de réaliser une enquête à indicateurs multiples sur le paludisme en 2021 à travers laquelle le taux d´utilisation des MILDA sera déterminé.
